# Influence of environmental temperature on chest compression quality and rescuer physiological responses

**DOI:** 10.1093/joccuh/uiaf065

**Published:** 2025-11-19

**Authors:** Taiki Miyazawa, Ginjiro Horiike, Ryosuke Yamauchi, Daisuke Ichikawa

**Affiliations:** Department of Sports and Physical Education, Faculty of Wellness, Shigakkan University, 55 Nakoyama, Yokonemachi, Obu-shi, Aichi 474-8651, Japan; Graduate School of Wellness, Shigakkan University, 55 Nakoyama, Yokonemachi, Obu-shi, Aichi 474-8651, Japan; Graduate School of Wellness, Shigakkan University, 55 Nakoyama, Yokonemachi, Obu-shi, Aichi 474-8651, Japan; Department of Civil and Environmental Engineering, Faculty of Science and Engineering, Toyo University, 2100 Kujirai, Kawagoe-shi, Saitama 350-8585, Japan

**Keywords:** cardiopulmonary resuscitation (CPR), chest compressions, heat stress, rescuer fatigue, thermoregulation

## Abstract

**Objectives**: Performing chest compressions in high-temperature environments may increase the physiological strain on rescuers, potentially affecting the quality of cardiopulmonary resuscitation (CPR). This study aimed to examine the effects of environmental temperature on physiological responses, perceived exertion, and chest compression performance.

**Methods**: Twenty male participants performed 10 minutes of chest compressions under 2 temperature conditions: 35°C (HOT) and 25°C (CON). Physiological parameters, including rectal temperature (*T*_re_), skin temperature, heart rate (HR), and rating of perceived exertion (RPE), were measured. The number of successful chest compressions was also recorded.

**Results**: The results indicated no significant differences in *T*_re_, heart rate, or RPE during chest compressions between HOT and CON. However, *T*_re_ showed a tendency to increase continuously during the recovery phase at 35°C. HR decreased during recovery in both conditions but remained elevated at 35°C compared with resting levels. A significant interaction effect was observed in mean skin temperature, suggesting different thermoregulatory responses between HOT and CON.

**Conclusions**: These findings suggest that although subjective exertion and cardiovascular responses may not differ significantly, prolonged exposure to high temperatures could influence thermoregulation in rescuers. Future research should explore the long-term impact of heat stress on CPR performance and rescuer safety.

## Teaser

Key points

**What is already known on this topic:** Chest compressions during cardiopulmonary resuscitation (CPR) are physically demanding and can be affected by environmental stressors. Previous studies have shown that extreme heat (≥40°C) may impair CPR quality and increase physiological burden. However, limited data exist regarding moderate high-temperature conditions (eg, 35°C) that are more reflective of real-world emergency scenarios in countries like Japan.

**What this study adds:** This study demonstrates that even at 35°C, a realistic summer temperature in Japan, the number of successful chest compressions decreases and physiological strain—particularly core temperature and heart rate—remains elevated during recovery. Notably, these effects occurred despite no significant differences in perceived exertion or core temperature during chest compressions.

**How this study might affect research, practice, or policy:** These findings suggest that even moderate heat stress can subtly degrade CPR performance. Future research and emergency preparedness policies should consider environmental temperature as a key factor influencing rescuer performance and safety. Cooling interventions or modified CPR protocols may be needed in hot environments.

## Background

Cardiopulmonary resuscitation (CPR) is a vital life-saving procedure performed in emergency situations to maintain circulation and oxygen delivery in individuals experiencing cardiac arrest. The quality of chest compressions, defined by appropriate depth, rate, and minimal interruptions, is a critical factor in determining the overall effectiveness of CPR.^1^^,^^2^ However, performing chest compressions is physically demanding, and the accumulation of fatigue can compromise CPR quality over time.^3^^,^^4^ Fatigue in rescuers can be influenced by various factors, including environmental conditions such as ambient temperature.

In real-world emergency settings, rescuers may be required to perform CPR under extreme environmental conditions, especially during summer. High temperatures have been reported to raise core temperature, heart rate (HR), and perceived exertion (RPE), leading to an increased physiological burden and a decline in physical performance.^5^^,^^6^ For example, Riva et al^7^ showed that extreme heat (40°C) reduces chest compression depth and speed while increasing rescuer fatigue, ultimately decreasing CPR effectiveness. Similarly, a crossover trial by Martin-Conty et al^8^ reported elevated HR and core temperature during CPR under thermal stress (above 40°C), with CPR quality deteriorating over time. However, most previous studies may not fully reflect the challenges of real-life rescue operations. Additionally, few studies have directly compared CPR performance between high (35°C) and moderate (25°C) ambient temperatures, particularly under conditions representative of Japan’s hot and humid summer. In Japan, emergency medical services frequently operate in environments exceeding 35°C with high humidity, which can pose serious risks to both rescuer safety and CPR performance. For example, during the summer months, the number of “mousho-bi” days (≥35°C) has increased nationwide, with an annual average of approximately 3.0 days between 1995 and 2024, and major cities such as Tokyo and Nagoya frequently record temperatures above 35°C during outdoor events.^9^^,^^10^ According to the Ministry of Health, Labour and Welfare, 1257 occupational heat-stroke cases were reported in 2024, highlighting the increasing risk of heat-related incidents in outdoor work and emergency settings.^11^ In such conditions—for example, outdoor festivals, sporting events, construction sites, and disaster rescue operations—rescuers may need to perform CPR in environments with both high ambient temperatures and humidity, which can amplify physiological strain.

This study aimed to address these gaps by simulating environmental conditions reflective of a typical Japanese summer and assessing their impact on chest compression performance. Specifically, we compared rectal temperature (*T*_re_), skin temperature, HR, RPE, the number of successful chest compressions, and body weight loss under 2 ambient temperature conditions (35°C and 25°C). We hypothesized that chest compression performance would deteriorate and physiological strain would increase under the higher temperature condition. The findings of this study are expected to provide practical insights for optimizing CPR protocols in hot environments and improving rescuer safety during prehospital care.

## Methods

### Participants

Twenty healthy men (mean ± SD: age 21 ± 2 years, height 173 ± 6 cm, body mass 66 ± 7 kg, body mass index [BMI] 22 ± 2 kg/m^2^) voluntarily participated in this study. All participants had completed CPR training, including chest compressions, within the past 3 years. Written informed consent was obtained from each participant following a detailed explanation of the procedures and potential risks. The study was approved by the Institutional Review Board of the Faculty of Wellness at Shigakkan University (IRB #273) and complied with the Declaration of Helsinki. None of the participants were taking medications that could influence cardiovascular responses. All were familiarized with the equipment and procedures before testing.

### Experimental design

A crossover design was used in which each participant underwent 2 experimental trials under different environmental conditions: HOT (35°C) and CON (25°C), both with 50% relative humidity, in a climatic chamber (TBR-6W2S2L2M; Espec Co, Osaka, Japan). These conditions simulated a typical Japanese summer (HOT) and a moderate environment (CON). Trial order was randomized, and each session was conducted at the same time of day to control for circadian variation. A minimum 1-week interval was set between sessions to avoid carryover effects and ensure participant recovery.

### Protocols

All experiments were conducted in the morning during winter. Participants consumed a standardized light breakfast consisting of a Calorie Mate Block (400 kcal; 8.2 g protein, 22.2 g fat, 43.4 g carbohydrate; Otsuka Pharmaceutical Co, Ltd, Tokyo, Japan) and 280 mL of water by 08:00 am. They arrived at the laboratory by 10:00 am, having abstained from caffeine, alcohol, and strenuous physical activity for at least 24 hours. Upon arrival, they drank 200 mL of water and sat for 30 minutes to stabilize fluid balance. They were weighed nude, changed into a provided shirt and shorts, inserted a rectal thermistor 10 cm deep for *T*_re_ measurement, and entered the climatic chamber. Participants sat for 1 hour without backrests while instruments were attached. After 15 minutes of resting data collection (REST), they assumed a chest compression posture within 30 seconds and performed compressions for 10 minutes (CC), reflecting the average emergency response time in Japan. A CPR manikin (Little Anne QCPR; Leardal Medical Co) placed on the floor was used, and compressions followed a metronome pace of 110 per minute (the recommended rate in the Japanese guidelines). Afterward, participants returned to a seated position within 30 seconds and recovered for 15 minutes (REC). Finally, they exited the chamber, wiped off the sweat using a towel, and were weighed nude again to determine body weight loss.

### Measurements

*T*_re_ was measured using a rectal thermistor (model 402 J; NIKKISO-THERM Co, Tokyo, Japan) inserted into the rectum to a depth of 10 cm from the anal verge, as marked by a piece of adhesive tape placed on the probe beforehand to ensure consistent insertion depth across participants. After insertion, the probe was further secured externally with surgical tape to prevent displacement throughout the experimental session. Skin temperature was measured using a surface-type thermistor probe (model 409 J; NIKKISO-THERM Co, Tokyo, Japan), which is designed such that only the contact surface with the skin contains the temperature-sensing element. According to the manufacturer’s specifications, the device ensures high measurement accuracy with an error margin of ±0.1°C under ambient conditions ranging from 20°C to 40°C and relative humidity of 20%-80%. However, the manufacturer does not indicate the corresponding JIS (Japanese Industrial Standards) accuracy classification (eg, Class A or B) for this product in the official documentation. Given that the experimental environment was well controlled within these specified ranges, and that the sensors were securely attached to the skin throughout the protocol, the influence of ambient air on skin temperature readings was considered minimal. The skin temperature probes were placed on the right side of the upper arm (*T*_arm_), chest (*T*_chest_), thigh (*T*_thigh_), and leg (*T*_leg_). The site of probe placement was identical for each participant. The mean skin temperature (*T*_sk_) was calculated using Ramanathan’s formula^12^:


(1) \begin{align*} T_{sk}=0.3\times(T_{\mathrm{arm}}+T_{\mathrm{chest}})+0.2\times(T_{\mathrm{thigh}}+T_{\mathrm{leg}}). \end{align*}

HR was measured by electrocardiography. *T*_re_, skin temperature, and HR data were continuously sampled at 1 kHz using an analog-to-digital converter (PowerLab; AD Instruments, Milford, MA, USA) interfaced with a computer. For analysis, average values from the last minute of each phase—REST, CC, and REC—were used.

The RPE data were recorded at the end of each phase using a 6-20 point scale. The CPR mannequin provided real-time feedback, and the number of successful compressions (depth >5 cm) was recorded via a QCPR application (QCPR; Leardal Medical Co, Stavanger, Norway) on a tablet. Body weight loss was calculated as the difference in nude weight before and after the experiment.

### Statistical analysis

Statistical analysis was performed using SPSS 25 (SPSS, Chicago, IL, USA). Following confirmation of distribution normality using the Shapiro-Wilk tests, a 2-way (Temperature: CON and HOT) × (Phase: REST, CC, and REC) repeated measures analysis of variance (ANOVA) with Bonferroni post hoc corrections was applied to *T*_re_, *T*_sk_, HR, and RPE. Student *t* tests compared successful compression counts and weight loss between HOT and CON. Data are expressed as mean ± SD, with significance for all tests set at *P* < .05.

## Results

**Figure 1:**
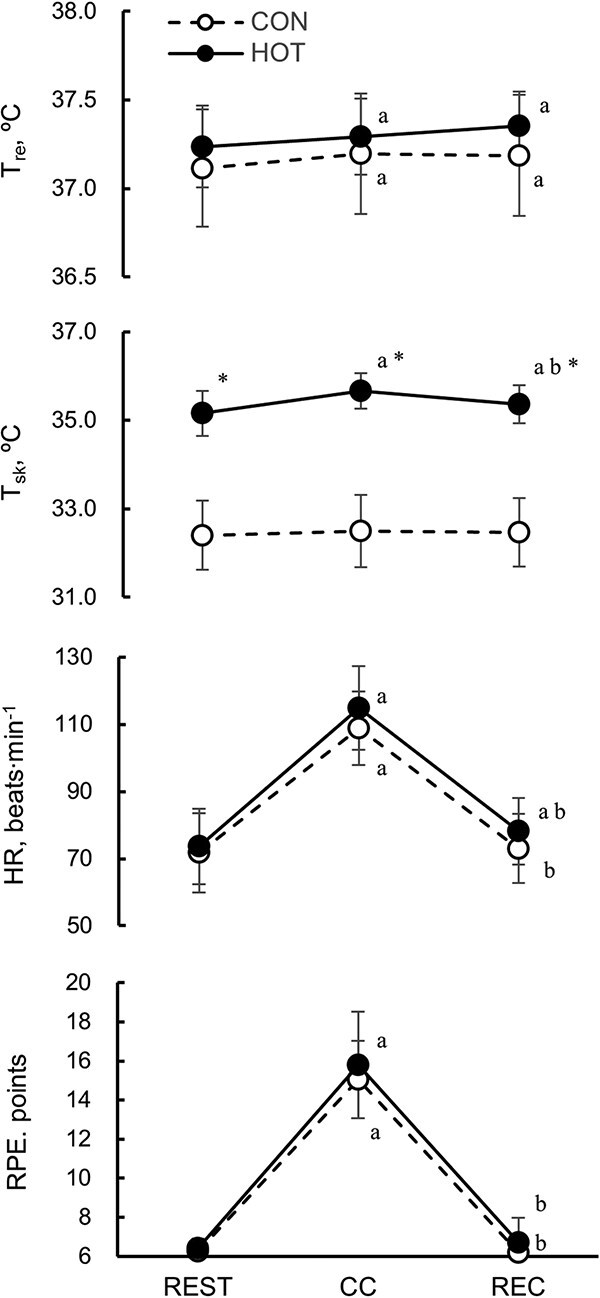
Rectal temperature (*T*_re_), mean skin temperature (*T*_sk_), heart rate (HR), and rating of perceived exertion (RPE) in the CON (white circle) and HOT (black circle) at the end of each phase (REST, CC, and REC). Values are presented as the mean ± SD for 20 participants. a, b, Denote significant difference (*P* < .05) compared with REST and CC, respectively. ^*^Denotes significant difference (*P* < .01) compared with CON. CC, chest compression phase; CON, 25°C ambient temperature; HOT, 35°C ambient temperature; REC, recovery phase; REST, resting phase.

A 2-way repeated measures ANOVA was conducted to examine the effects of environmental temperature (CON and HOT) and phase (REST, CC, and REC) on *T*_re_, *T*_sk_, HR, and RPE. A significant interaction was found in *T*_sk_ (f = 8.04, *P* < .01). The main effect of temperature was significant (f = 229.43, *P* < .01) only in *T*_sk_. Furthermore, the main effect of phase was significant in *T*_re_ (f = 17.40, *P* < .01), *T*_sk_ (f = 16.76, *P* < .01), HR (f = 392.39, *P* < .01), and RPE (f = 456.91, *P* < .01) (Table  1). The results of post hoc analyses using Bonferroni correction are shown below (Table  1 and Figure 1).

**Table 1:** The 2-way repeated measures ANOVA of *T*_re_, *T*_sk_, HR, and RPE.

**Variables**	**Temperature**	**Phase**			***P* value of ANOVA**		
		**REST (95% CI)**	**CC (95% CI)**	**REC (95% CI)**	**Interaction (temperature × phase)**	**Main effect**	
						**Temperature**	**Phase**
*T*_re_, °C	CON	37.11 ± 0.33 (36.96-37.27)	37.19 ± 0.34^a^ (37.04-37.35)	37.19 ± 0.34^a^ (37.03-37.35)	.11	.15	<.01
	HOT	37.24 ± 0.23 (37.13-37.34)	37.29 ± 0.22^a^ (37.19-37.39)	37.35 ± 0.19^a^ (37.26-37.44)			
*T*_sk_, °C	CON	32.40 ± 0.78 (32.04-32.77)	32.50 ± 0.81 (32.12-32.88)	32.46 ± 0.77 (32.10-32.83)	<.01	<.01	<.01
	HOT	35.15 ± 0.51^*^ (34.92-35.39)	35.66 ± 0.40^a^^*^ (35.47-35.85)	35.36 ± 0.43^a^^b^^*^ (35.16-35.56)			
HR, beats/min	CON	71.7 ± 11.9 (66.2-77.3)	108.9 ± 10.9^a^ (103.8-114.0)	73.0 ± 10.4^b^ (68.2-77.9)	.40	.16	<.01
	HOT	73.7 ± 11.3 (68.4-78.9)	114.9 ± 12.5^a^ (109.0-120.7)	78.1 ± 10.0^a^^b^ (73.5-82.8)			
RPE, points	CON	6.3 ± 0.4 (6.0-6.5)	15.1 ± 2.0^a^ (14.1-16.0)	6.2 ± 0.4^b^ (6.0-6.4)	.68	.08	<.01
	HOT	6.4 ± 0.5 (6.2-6.6)	15.8 ± 2.7^a^ (14.5-17.9)	6.7 ± 1.3^b^ (6.1-7.3)			

Abbreviations: ANOVA, analysis of variance; CC, chest compression phase; CON, 25°C ambient temperature; HOT, 35°C ambient temperature; HR, heart rate; REC, recovery phase; REST, resting phase; RPE, rating of perceived exertion; *T*_re_, rectal temperature; *T*_sk_, mean skin temperature.

Values are presented as mean ± SD for 20 participants.

a Denotes significant difference (*P* < .05) compared with REST.

b Denotes significant difference (*P* < 0.05) compared with CC.

* Denotes significant difference (*P* < .01) compared with CON.

*T*_re_ during CC and REC was significantly (*P* < .05) increased compared with REST in both CON and HOT. In HOT, *T*_re_ remained elevated after CC and tended to be higher (*P* = .06) at REC than at CC.

*T*_sk_ in HOT was significantly (*P* < .01) higher than in CON at all phases (REST, CC, and REC). In HOT, *T*_sk_ at CC (*P* < .01) and REC (*P* < .05) were significantly higher compared with REST. After chest compressions, *T*_sk_ decreased in HOT, and there was a significant difference between CC and REC (*P* < .01). In CON, there were no significant changes in *T*_sk_ between all phases.

HR during CC in both CON and HOT was significantly (*P* < .01) higher than at REST. At REC, HR in CON and HOT was significantly (*P* < .01) lower than at CC. In HOT, HR during REC remained significantly (*P* < .05) higher than at REST.

In both CON and HOT, RPE was significantly (*P* < .01) higher at CC than at REST, and lower at REC than at CC.

**Figure 2:**
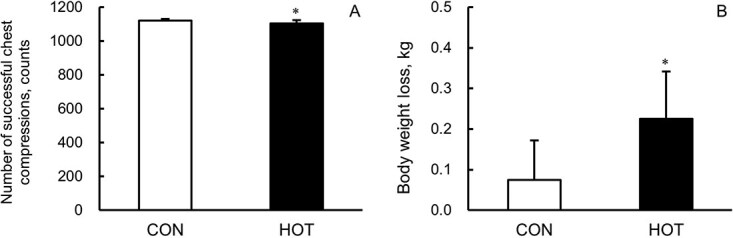
Number of successful chest compressions (A) and body weight loss (B) in the CON (white) and HOT (black). Values are presented as the mean ± SD for 20 participants. *Denotes significant difference (*P* < .01) compared with CON. CON, 25°C ambient temperature; HOT, 35°C ambient temperature.

The number of successful chest compressions was significantly (*P* < .01) lower in HOT (1105 ± 18 counts [95% CI, 1096-1113]) than CON (1120 ± 10 counts [95% CI, 1116-1125]). The effect size (Cohen *d*) was 0.83, which corresponds to a large effect (Figure 2A).

Body weight loss was significantly greater (*P* < .01) in HOT (0.22 ± 0.12 kg [95% CI, 0.17-0.28]) than in CON (0.08 ± 0.10 kg [95% CI, 0.03-0.12]). The effect size (Cohen *d*) was 0.99, which corresponds to a large effect (Figure 2B).

## Discussion

This study examined the effects of different environmental temperatures (35°C and 25°C) on chest compression performance and physiological burden. *T*_re_, HR, and RPE during compressions showed no significant differences between HOT and CON. However, HOT resulted in fewer successful compressions and greater body weight loss. During REC, *T*_re_ continued to increase in HOT, and HR remained elevated compared with REST. These findings suggest that the environmental temperature influenced chest compression performance.

In this study, *T*_re_ increased significantly from the resting state due to chest compression in HOT. An increase in core temperature is considered a limiting factor for exercise performance, with a marked decline observed when the core temperature exceeds 38.5°C.^13-15^ Since *T*_re_, an index of core temperature, did not reach 38°C in this study, it is unlikely that the increase in *T*_re_ significantly affected the performance of chest compressions over the 10-minute period.

The HR during CC in HOT was 114.9 ± 12.5 beats/min in the present study (Table  1). Given that the estimated maximum HR (HR_max_ = 220 – age) for participants (21 ± 2 years) is approximately 199 beats/min, the exercise intensity during chest compressions was calculated as approximately 57.7% HR_max_. Furthermore, using the resting HR (73.7 ± 11.3 beats/min), the exercise intensity calculated based on HR reserve (HRR) was estimated at 32.9% HRR. Therefore, the chest compressions performed in this study corresponded to low-to-moderate intensity exercise. Previous studies demonstrated that the moderate intensity exercise (60%-70% maximum oxygen uptake [VO_2max_]) lasted for approximately 30-50 minutes in a hot environment (40°C).^5^^,^^16^ Given that the chest compression task in this study was performed at an intensity that was comparable to, or even lower than, the intensities reported in previous studies and under a lower ambient temperature (35°C rather than 40°C), it is plausible that chest compressions at this intensity can be sustained for at least 10 minutes even in a high-temperature environment.

Dehydration due to sweating is one of the factors that limit exercise performance.^17^^,^^18^ A body weight loss exceeding 2% has been shown to impair endurance performance and cognitive/mental performance, which is crucial in tasks requiring concentration, skilled execution, and decision-making.^17^ In the present study, body weight loss, an index of sweating, was significantly higher in the 35°C environment. However, as this corresponded to only approximately 0.3% of the participants’ average body weight (66 ± 7 kg), the effect of dehydration on chest compression performance is likely to be minimal.

In HOT, *T*_re_ exhibited an increasing trend during the recovery phase (REC) compared with the CC (*P* = .059), and HR remained elevated compared with the resting state (*P* < .05). These results suggest that the physiological burden induced by chest compressions persists for at least 15 minutes post-exercise. Heat loss is suppressed when the temperature difference between the skin and the environment is small.^19^ In this study, because the temperature difference was smaller in HOT than in CON, heat loss was likely inhibited, resulting in a slower decline in *T*_re_ during REC. It should also be noted that *T*_re_ typically shows a delayed response compared with other core temperature indices, such as esophageal or gastrointestinal temperatures.^20-22^ This delayed responsiveness may have contributed to the continued rise in *T*_re_ during REC under the HOT condition. Additionally, *T*_sk_ remained significantly higher in HOT (*P* < .01), indicating increased cutaneous vasodilation and skin blood flow.^19^^,^^23^ Furthermore, body weight loss, indicating fluid loss, was also greater in HOT. These factors may contribute to the sustained elevation in HR during REC. Further research is needed to clarify the mechanisms behind elevated *T*_re_ and HR in HOT recovery.

In this study, the number of successful chest compressions was significantly lower in HOT than in CON. *T*_re_ and HR during CC showed no significant differences, and the increase in body weight loss was relatively small. These factors likely had limited influence, indicating other contributors. Prior studies suggest that high temperatures accelerate muscle fatigue.^24^ Although RPE did not differ between conditions, psychological and cognitive factors may have affected performance. Heat has been shown to impair cognition and concentration,^25^^,^^26^ decrease motivation,^27^ and increase psychological stress due to thermal discomfort.^28^ Further research is needed to explore how these factors contribute to decreased compression performance in hot environments.

This study has several limitations. First, as only young males participated, effects on females or older adults remain unknown. Given that sex and age influence thermoregulatory and cardiovascular responses to exercise and heat,^29^^,^^30^ the results cannot be generalized to broader populations. Second, chest compressions were limited to 10 minutes, so responses during prolonged CPR are unclear. Previous research has shown that longer durations of exercise increase participants’ body temperature and fatigue, especially under heat stress.^31^^,^^32^ Third, the controlled indoor setting may not reflect real-world rescues, where additional heat stress from direct sunlight (radiant load) and environmental unpredictability can worsen physiological strain. Environmental factors such as solar radiation and wind have been shown to substantially influence thermoregulatory responses during physical tasks.^33^^,^^34^ Future studies should include more diverse participants and replicate actual rescue environments under variable climatic conditions.

## Conclusions

The findings of this study indicate that high ambient temperatures (35°C) significantly reduce the number of successful chest compressions and increase body weight loss. Additionally, during the recovery phase, *T*_re_ remained elevated, and HR stayed above resting levels, highlighting the importance of heat stress management in high-temperature rescue operations. Future research should focus on developing specific intervention strategies to prevent performance decline in rescuers.

## Data Availability

The datasets from this study are available from the corresponding author upon reasonable request.
